# Supplementary material for: Cadherin Expression Profiles Define Glioblastoma Differentiation and Patient Prognosis
Source: Cancers (Basel). 2024 Jun 22;16(13):2298. doi: 10.3390/cancers16132298 (PMC11240393; doi:10.3390/cancers16132298)
Supplement: Supplementary file 1 [file cancers-16-02298-s001.zip › cancers-3062201-supplementary.pdf]

Cadherin Expression Profiles Define Glioblastoma Differentiation and Patient Prognosis

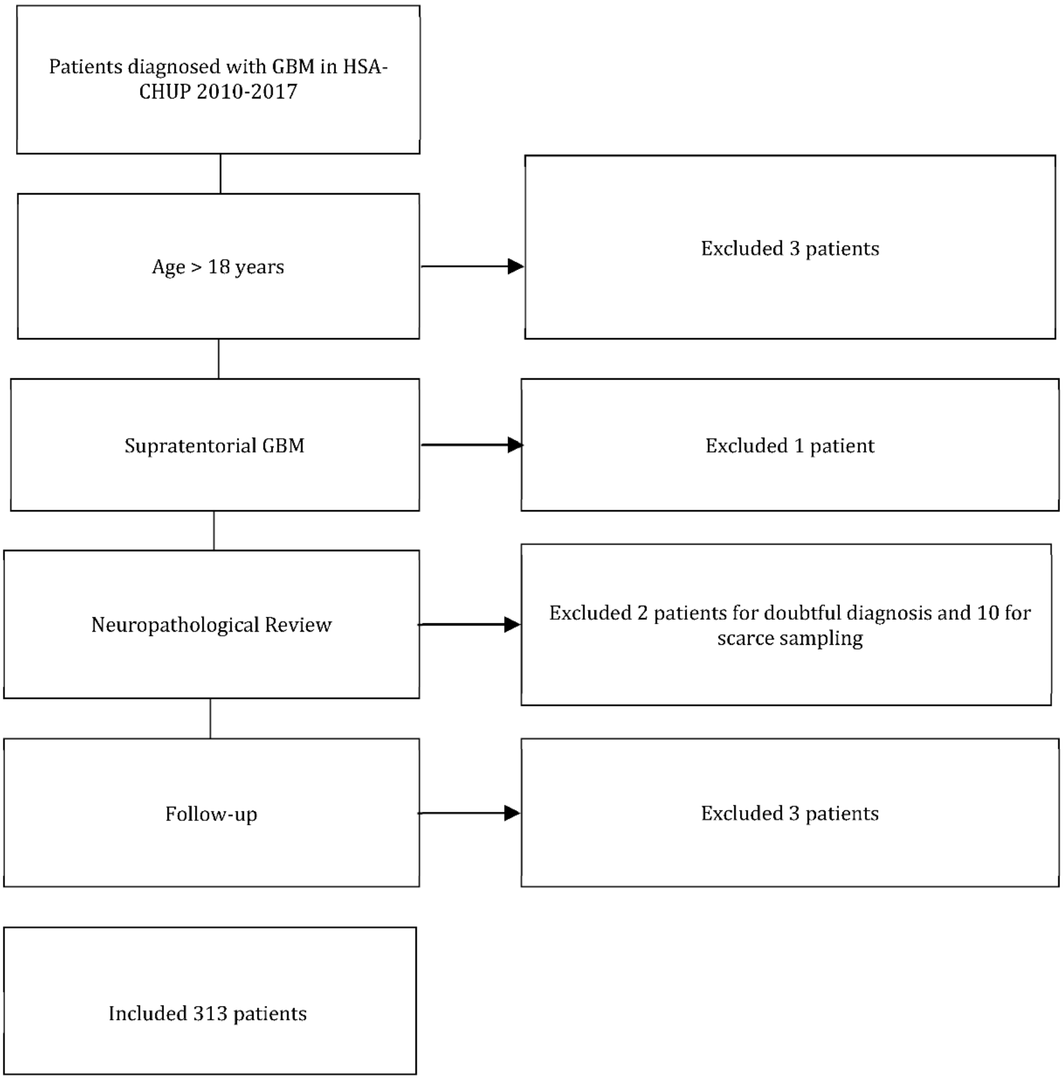

Supplementary Figure S1. Scheme representing patient inclusion algorithm to the selected cohort of glioblastomas.

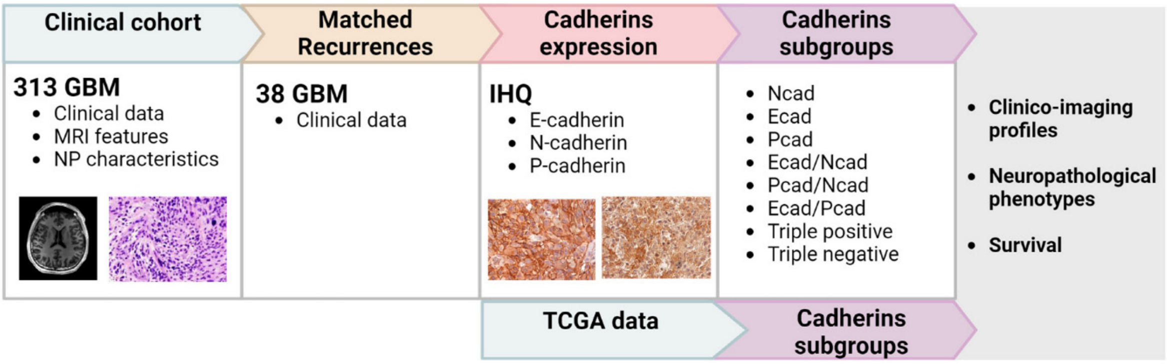

Supplementary Figure S2. Study design.

Supplementary Table S1. Classification algorithm used to evaluate cadherins expression in GBM, taking in consideration staining intensity, staining extension and membrane positivity.

Intensity	Extension	Membrane
+ (1 point)	<25% (1 point)	no (1 point)
++ (2 points)	25-50% (2 points)	yes (2 points)
+++ (3 points)	>50% (3 points)	

Supplementary Table S2. Characterization of glioblastoma series.

**Gender**	
Male	178 (56,9%)
Female	135 (43,1%)
**Age (mean)**	61,743 ± 10,483
**Presentation**	
Behaviour	66 (21,1%)
Focal deficit	126 (40,3%)
Headache	65 (20,8%)
Seizure	52 (16,6%)
**Initial functional status (KPS) (mean)**	79,33 ± 10,585
**MRI characteristics**	
Major axis (mean)	7,27 ± 2,09 cm
Location	Frontal (36,1%)<br>Temporal (30,4%)<br>Parietal (22,4%)
Multilobar	71 (22,7%)
Ependymal extension	89 (28,4%)
Corpus callosum extension	92 (29,4%)
Multifocality	76 (24,3%)
Cysts	49 (15,7%)
**Treatment**	
Surgery	177 (56.5%)
Biopsy	136 (43.5%)
Radiotherapy	19 (6,1%)
Radiotherapy + Chemotherapy	8 (2,5%)
Stupp protocol	189 (60,4%)
**Recurrence**	297 (94,89%)
2nd line treatments	159 (53,5%)
Re-operated	38 (12,79%)
